# Temporal assessment of nanoparticle accumulation after experimental brain injury: Effect of particle size

**DOI:** 10.1038/srep29988

**Published:** 2016-07-22

**Authors:** Vimala N. Bharadwaj, Jonathan Lifshitz, P. David Adelson, Vikram D. Kodibagkar, Sarah E. Stabenfeldt

**Affiliations:** 1 School of Biological and Health Systems Engineering, Arizona State University, Tempe, AZ, USA; 2 Barrow Neurological Institute at Phoenix Children’s Hospital, Phoenix, AZ, USA; 3 Department of Child Health, University of Arizona, College of Medicine-Phoenix, Phoenix, AZ, USA.

## Abstract

Nanoparticle (NP) based therapeutic and theranostic agents have been developed for various diseases, yet application to neural disease/injury is restricted by the blood-brain-barrier (BBB). Traumatic brain injury (TBI) results in a host of pathological alterations, including transient breakdown of the BBB, thus opening a window for NP delivery to the injured brain tissue. This study focused on investigating the spatiotemporal accumulation of different sized NPs after TBI. Specifically, animal cohorts sustaining a controlled cortical impact injury received an intravenous injection of PEGylated NP cocktail (20, 40, 100, and 500 nm, each with a unique fluorophore) immediately (0 h), 2 h, 5 h, 12 h, or 23 h after injury. NPs were allowed to circulate for 1 h before perfusion and brain harvest. Confocal microscopy demonstrated peak NP accumulation within the injury penumbra 1 h post-injury. An inverse relationship was found between NP size and their continued accumulation within the penumbra. NP accumulation preferentially occurred in the primary motor and somatosensory areas of the injury penumbra as compared to the parietal association and visual area. Thus, we characterized the accumulation of particles up to 500 nm at different times acutely after injury, indicating the potential of NP-based TBI theranostics in the acute period after injury.

Traumatic brain injury (TBI) is a leading cause of disability worldwide^1^ with 1.7 million TBIs reported annually in the United States^2^. The pathology of TBI occurs from both immediate and delayed mechanisms resulting in highly heterogeneous tissue damage^3^. This pathology may include substantial blood-brain-barrier (BBB) dysfunction due to alterations in the capillary endothelial cells, specifically deregulation of tight junctions and/or vesicular transport^4^. As the BBB breaks down, normally impermeable blood constituents may now freely extravasate into the brain parenchymal space^5^. This transient, increased permeability within the injury penumbra may offer a unique opportunity to deliver drugs that are normally excluded from the brain. In order to exploit this potential avenue for delivery after TBI, further characterization of the temporal profile of the “permeability window” as well as the size range for molecule/particle extravasation is necessary.

In laboratory settings, experimental animal brain trauma models provide insights into the events that occur during and after injury. One of the most commonly used models is the controlled cortical impact (CCI) model in the rodent; this model produces focal damage leading to major cortical damage directly in the zone of impact^6^. Previous CCI model studies with rats established the presence of a compromised BBB as indicated by the extravasation of horseradish peroxidase (HRP)^6^ or Evans Blue (EB)^7^,^8^ post-injury. Specifically, the BBB was compromised immediately after injury and remained significantly permeable for 5–7 days post-injury within the injury penumbra (with a second peak at ~3 days)^6^,^7^. Furthermore, Habgood *et al*. used weight-drop injury model to demonstrate that large molecular weight (MW) markers (HRP ~40 kDa) were permeable up to 24 h post-injury, as compared to smaller MW markers (biotin-dextran-amine, <10 kDa) that remain permeable as late as 4 days post-trauma^9^. Therefore, the BBB, post-injury, displays variable permeability based on the MW, with equivalent hydrodynamic diameter of about 3–6 nm^10^,^11^. These seminal studies provided evidence of BBB dysfunction after TBI^6^,^7^,^9^; however, the dynamic size range for particle extravasation greater than ~10 nm has not been previously elucidated.

Nanoparticles (NPs), particles ranging from 10–1000 nm in diameter^12^, are utilized for various biomedical applications due to their pharmacological attributes. The unique physicochemical properties of NPs have shown promise in delivering a range of molecules, including water-insoluble drugs and large payloads, to desired sites in the body^13^,^14^,^15^,^16^. Specifically, surface modified NPs have been designed to achieve greater efficacy of therapeutic agents, prolonged pharmacological effects by improved drug protection, and reduced renal clearance compared to standard drug administration^17^,^18^,^19^. Moreover, contrast agents may be incorporated into NPs enabling visualization of the diseased site to diagnose and/or monitor the *in vivo* efficacy of the therapeutics^13^,^20^. However, these remarkable attributes of NPs are commonly unattainable for neural applications due to BBB permeability limitations. We postulate that the BBB disruption after TBI may afford a unique opportunity for NP delivery. Our hypothesis was recently supported by two independent studies demonstrating the feasibility and utility for intravenous NP delivery after TBI^21^,^22^. Yet, a systematic evaluation of the temporal window and the NP size range for NP delivery after TBI has not been previously performed. Therefore, the focus of this study was to establish the effect of NP size and time of NP injection after experimental TBI while maintaining a constant circulation time. As such, we investigated the accumulation of four different sized (20, 40, 100, and 500 nm) fluorescent polystyrene NPs at five time points acutely (up to 24 h) after TBI using the murine CCI model.

## Results

### Nanoparticle characterization

**Figure 1:**
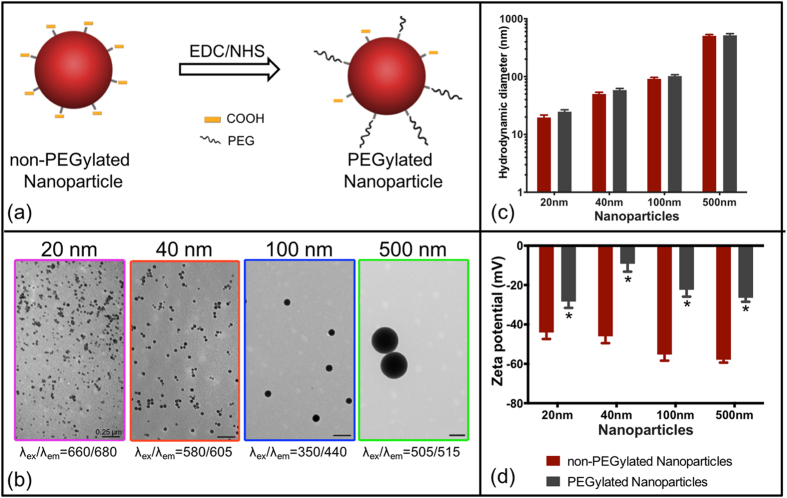
Nanoparticle characterization (**a**) Schematic of nanoparticle PEG conjugation using EDC/NHS chemistry. (**b**) TEM images of monodispersed nanoparticles (PEGylated). (**c**) Hydrodynamic diameter of non-PEGylated and PEGylated nanoparticles. (**d**) Zeta potential of non-PEGylated and PEGylated nanoparticles, *p < 0.05, t-test. Error bars represent standard error of mean with n = 3 per group.

Four sizes of carboxylated polystyrene NPs (20 nm, 40 nm, 100 nm and 500 nm), each internally loaded with a distinct fluorescent dye with negligible overlap in signal were employed in the study (Fig. 1). The surface of carboxylated NPs was modified with amine-polyethylene glycol (PEG)^23^,^24^ to reduce their zeta potential for improved NP stability and to prolong blood circulation time. The NPs were characterized via transmission electron microscopy (TEM) and dynamic light scattering (DLS). PEGylation of NPs via amine/carboxyl EDC/NHS chemistry was confirmed through DLS based on a decrease in zeta potential and modest increase (~10 nm) in hydrodynamic diameter of the NPs (Fig. 1d,c, Table 1; n = 3). A statistically significant decrease in zeta potential was observed for each NP after PEGylation (p < 0.05; Table 1, Fig. 1d). TEM images of NPs show monodispersed, spherical shaped particles for each population (Fig. 1b). To simplify nomenclature, the four NP groups employed in this study will be addressed by their nominal diameters, 20 nm, 40 nm, 100 nm, and 500 nm.

### *In vivo* study: Horseradish peroxidase (HRP) extravasation

**Figure 2:**
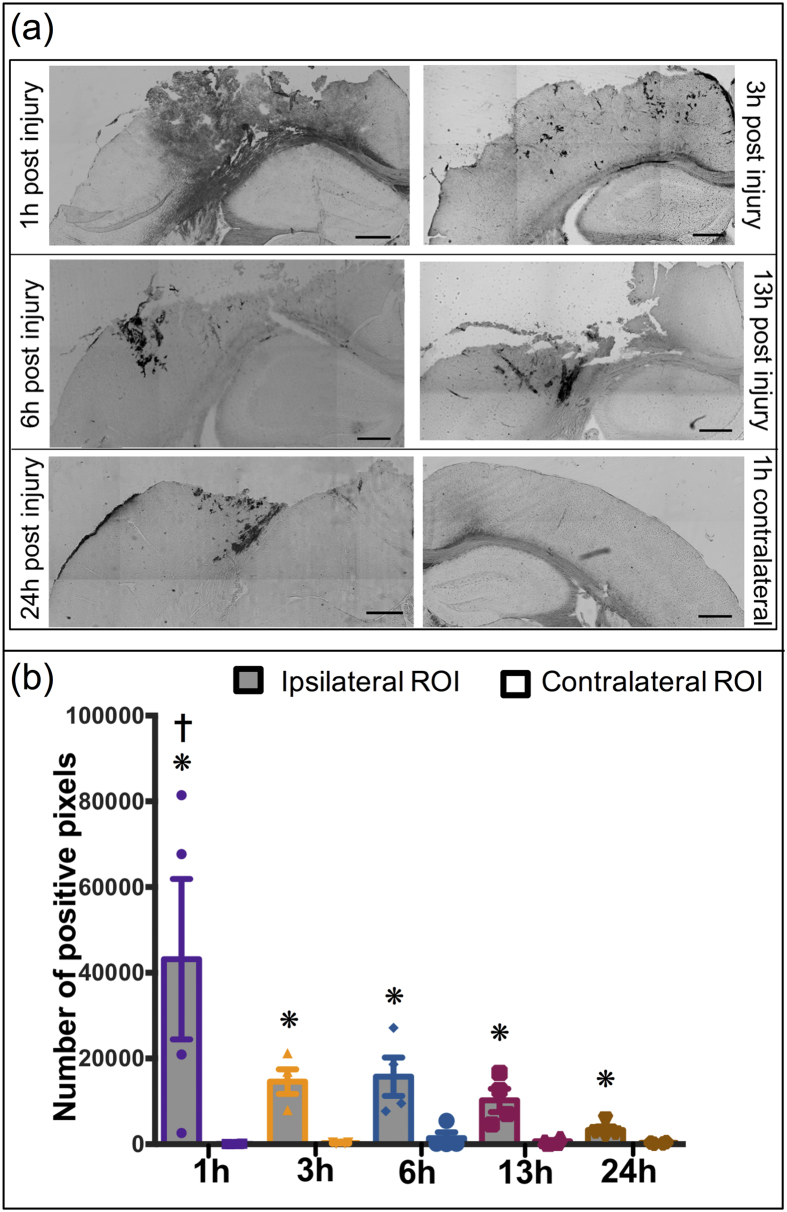
HRP Extravasation after TBI: (**a**) Representative images of extravasation of HRP injured region after 1 h, 3 h, 6 h, 13 h and 24 h post injury (**a–e**); contralateral region 1 h post injury (**f**). (**b**) Quantitative analysis of HRP extravasation over time. *p < 0.05 compared to their respective contralateral ROI, Student’s t-test. ^†^p < 0.05 compared to 13 h and 24 h ipsilateral ROI, Tukey’s *post-hoc* test. Error bars represents standard error of mean with n = 4 per group. Scale bar = 500 μm.

A lateral CCI was imparted on the frontoparietal cortex generating a cortical lesion ipsilateral to the impact and leaving the contralateral hemisphere uninjured. An intravenous retro-orbital^25^ HRP injection 10 min prior to sacrifice was included as a positive control to evaluate the BBB integrity, as extravasation of HRP is a well-accepted indicator of breached BBB^6^,^9^,^26^,^27^ (Supplementary Fig. 1). Specifically, we observed extravasation of HRP in the primary and the adjacent injury region at 1 h post injury. However, the HRP extravasation was only localized to the primary injury site at and after 3 h post injury. Therefore, the quantification of HRP staining for 1 h cohort included both the primary and adjacent tissue region; analysis for the remaining cohorts focused only the primary injury site. Quantification of HRP extravasation to obtain the number of positive pixels using ImageJ software, demonstrated significant differences between ipsilateral and contralateral locations (p = 0.0002) while a time dependent effect was not observed (p = 0.038) using two-way ANOVA. Pair-wise analysis of extravasation of HRP specifically for each time point revealed a significant increase in HRP extravasation in the injury penumbra compared to contralateral tissue at all investigated time points (1 h, 3 h, 6 h, 13 h and 24 h) post injury (p < 0.05; Fig. 2b). Comparing the ipsilateral HRP staining over time revealed a nearly 35% reduction in HRP staining at the 3 h, 6 h, 13 h, and 24 h time points compared to the maximal HRP staining at 1 h post-injury. This reduction was statistically significant for 13 h and 24 h cohorts (p < 0.05) compared to 1 h cohort, thus demonstrating potential resolution of the BBB over time. Therefore, HRP extravasation confirmed the BBB dysfunction up to 24 h post-injury corroborating previous studies^6^ (Fig. 2).

### *In vivo* study: Accumulation of nanoparticles within injury penumbra

**Figure 3:**
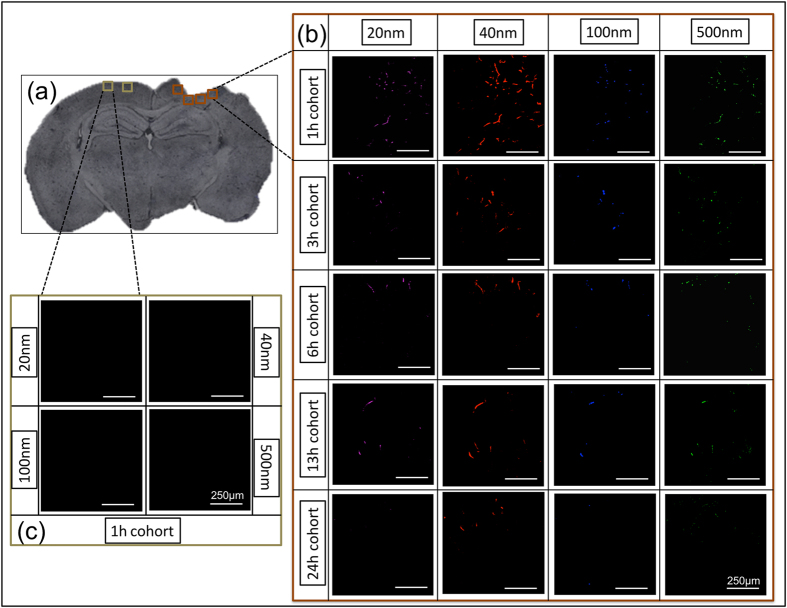
Accumulation of different size nanoparticles over time after injury. (**a**) Representative images of injured brain section (~−1.655 mm bregma, 25 μm thick). (**b**) Panel of 20X confocal images near the injury region on ipsilateral hemisphere (shown in (**a**)). Rows of the panel show time course and the columns show the different nanoparticle size. **(c)** Panel of 20X confocal images on contralateral hemisphere (shown in (**a**)), for each nanoparticle at 1 h post injury. Scale bar = 250 μm.

We used a NP cocktail containing particles with diameters ranging from 20 nm to 500 nm, to determine the extent of NP accumulation acutely (up to 24 h) after brain injury with a constant 1 h circulation time. Specifically, we quantified the accumulation of each fluorescent NP within processed brain tissue sections spanning across the injury lesion (~−0.18 mm bregma to ~−3.28 mm bregma) via confocal microscopy (Fig. 3). Interestingly, we observed maximum accumulation of all NPs 1 h after injury, including the 500 nm particles. Additionally, the results indicated prolonged NP accumulation of 20 nm and 40 nm up to 13 h post injury within the injury penumbra where as significant accumulation of 100 nm and 500 nm NPs was found up to 6 h.

**Figure 4:**
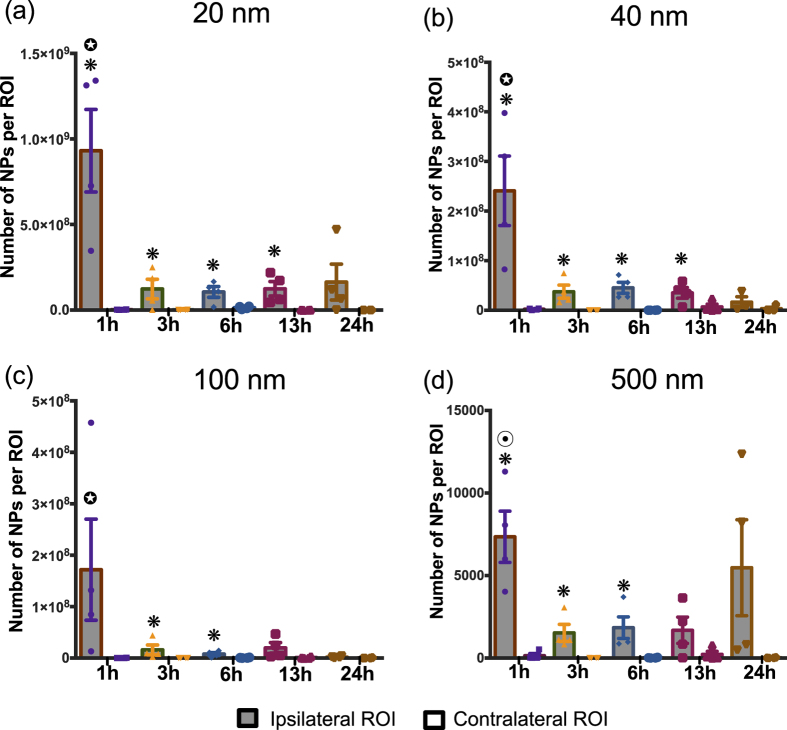
Nanoparticle accumulation after TBI. Accumulation of (**a**) 20 nm, (**b**) 40 nm, (**c**) 100 nm, (**d**) 500 nm nanoparticles at different time points after traumatic brain injury in mice. *p < 0.05 compared to their respective contralateral ROI, Student’s t-test. p < 0.05 compared to 3 h, 6 h, 13 h, and 24 h ipsilateral ROI, p < 0.05 compared to 3 h, 6 h, and 13 h ipsilateral ROI; Tukey’s *post-hoc* test. Error bars represents standard error of mean with n = 4 per group.

Two-way ANOVA results from our study revealed a significant difference between the ipsilateral and contralateral location for 20 nm (p < 0.0001), 40 nm (p < 0.0001), 100 nm (p = 0.0392) and 500 nm (p < 0.0001), (Fig. 4a–d). Moreover, the analysis demonstrated a significant time dependent effect for 20 nm, 40 nm, 100 nm and 500 nm (p = 0.0001), (p < 0.0001), (p = 0.043), (p = 0.0364) (Fig. 4a–d), respectively. To take a closer look at the effect of each of these variables individually, post-hoc pair-wise analyses of critical comparisons are described below.

#### Analysis of the BBB breach in injured and uninjured brain tissue

The first pairwise analysis focused on comparing NP accumulation within the ipsilateral injury penumbra to contralateral tissue at different time points (Fig. 4). Specifically, for 20 nm and 40 nm, ipsilateral accumulation markedly increased for all time points compared to contralateral tissue, except 24 h cohort (Fig. 4a,b; p < 0.05). For 100 nm, statistically significant increase in NP accumulation on the ipsilateral side was only observed at 3 h and 6 h post-injury (Fig. 4c; p < 0.05). Finally, the 500 nm NP accumulation was significantly greater for ipsilateral versus contralateral up to 6 h. Overall, 20 and 40 nm NPs significantly accumulated in the injury penumbra compared to the contralateral tissue up to 13 h after injury while the time window was reduced by nearly half (6 h) for the 100 and 500 nm.

#### Analysis of the BBB breach within injured region across different time points

The second critical pairwise analysis focused on comparing the temporal changes in NP accumulation within the ipsilateral injury penumbra across time points. For 20, 40 and 100 nm, there was a significant reduction in accumulation for 3 h, 6 h, 13 h and 24 h time points as compared to 1 h post injury (p < 0.05); less than 35% of 1 h NP accumulation was observed for other time points (Fig. 4a–c). Accumulation of the 500 nm NP was nearly 25% of 1 h cohort for 3 h, 6 h, and 13 h post-injury and was significantly reduced (p < 0.05; Fig. 4d). Interestingly, the mean NP accumulation for the 500 nm NP at 24 h exhibited similar accumulation as compared to the 1 h post injury; we noted that variance within this group was quite large as two of the four animals displayed high NP accumulation whereas the other two animals had modest NP accumulation. The overall trend for different sized NPs demonstrated maximum accumulation at 1 h post-injury compared to other time points.

### *In vivo* study: Spatial distribution of HRP and nanoparticles

**Figure 5:**
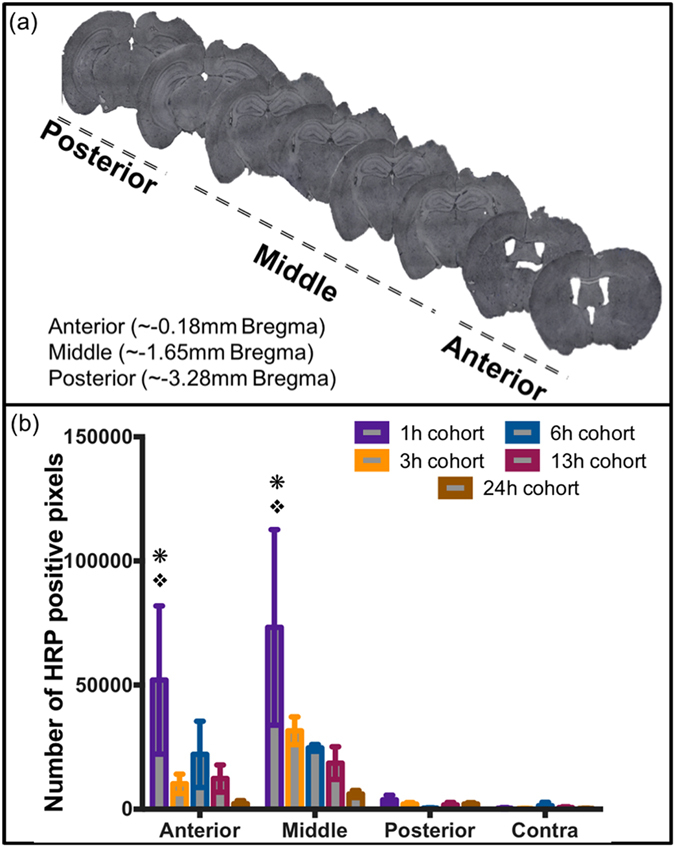
Spatial distribution analysis. (**a**) Representative brain images showing anterior, middle and posterior regions of the brain w.r.t. bregma. (**b**) Quantitative analysis of HRP extravasation at different anatomical regions and different time points after TBI. *p < 0.05 compared to contralateral ROI (Contra), p < 0.05 compared to posterior ROI (Posterior); Tukey’s *post-hoc* test. Error bars represents standard error of mean with n = 4 per group.

**Figure 6:**
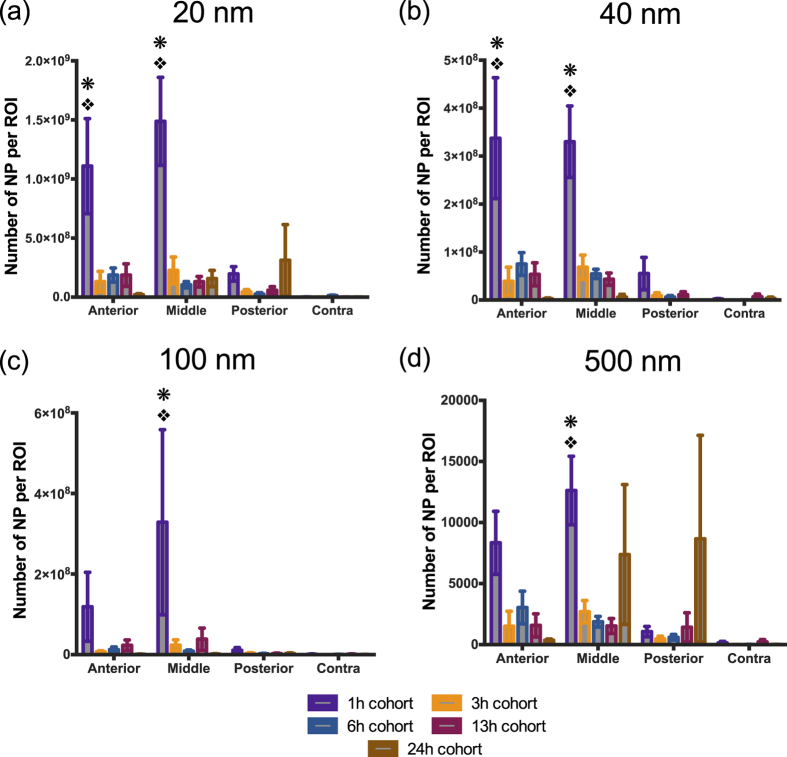
Spatial distribution of nanoparticle accumulation. Quantitative analysis of (**a**) 20 nm, (**b**) 40 nm, (**c**) 100 nm, (**d**) 500 nm nanoparticles at different anatomical regions and different time points. *p < 0.05 compared to contralateral ROI (contra), p < 0.05 compared to posterior ROI (posterior); Tukey’s *post-hoc* test. Error bars represents standard error of mean with n = 4 per group.

The CCI impact to the frontoparietal cortex (−1.5 mm bregma, 1.5 mm lateral from midline) generates an injury lesion mainly to the cortex, which includes damage to the primary motor area (M1), primary somatosensory area, posterior parietal association area and anteriomedial visual cortex (V1). Interestingly, we observed selective distribution of HRP and NP accumulation based on the cortical region, a trend that held consistent with all post-injury time point cohorts. In this regard, we examined a series of coronal sections across the injury lesion from anterior to posterior. We binned the tissue sections into three spatial sub-groups: anterior (~−0.18 mm bregma), middle (~−1.65 mm bregma) and posterior (~−3.28 mm bregma) (Fig. 5a). Our results demonstrated maximal accumulation of the NPs and HRP within the anterior and middle regions of the injury penumbra (statistically significant only at 1 h), as compared to that of posterior and contralateral regions (Figs 5 and 6). Strikingly, the accumulation in the posterior region was similar to that of the contralateral region.

HRP extravasation significantly varied across the injury penumbra (p = 0.0003) (Fig. 5b). Further pairwise post-hoc analysis revealed a significant increase in extravasation for 1 h post-injury within the anterior and middle injury penumbra regions, compared to both posterior injury penumbra, and contralateral tissue. Furthermore, HRP extravasation within the posterior injury penumbra was not significantly different than contralateral tissue.

Significant difference in accumulation was observed across the different regions of the brain for 20 nm, 40 nm, and 500 nm (p = 0.0002), (p < 0.0001), (p = 0.02), respectively yet not significant for 100 nm (p = 0.10), (Fig. 6). Tukey’s post-hoc analysis of the 20 nm and 40 nm NPs demonstrated a significant increase in accumulation for the anterior injury region at 1 h compared to both the posterior injury region, and contralateral tissue (p < 0.05) (Fig. 6a,b). The accumulation of 20 nm, 40 nm, 100 nm and 500 nm NP within the core of the injury penumbra was significantly more than the posterior injury penumbra, and contralateral tissue at 1 h post injury (p < 0.05) (Fig. 6a–d). Interestingly, no significance was observed in NP accumulation between the posterior injury penumbra and the contralateral tissue regardless of the NP size.

## Discussion and Conclusion

Theranostic delivery for the brain has been largely hindered by limitations of BBB permeability. However, short windows of BBB dysfunction or damage as a result of disease or injury pathology may provide an opportunity for delivery of contrast agents and poorly soluble drugs via NPs. To fully utilize the window of opportunity of BBB opening that occurs after TBI, we need to further assess the spatiotemporal accumulation of NPs after injury. The study presented here directly addresses the critical knowledge gap to determine to effect of different size NP accumulation and the injection time points after experimental TBI, where the results provide key insights into NP behavior after TBI. Specifically, three key findings include: 1. NPs up to 500 nm may be delivered to TBI injured brain, 2. Maximal NP accumulation occurs 1 h after TBI, 3. NP accumulation of 20 nm and 40 nm NPs occurred out to 13 h post-injury.

Systemic NP delivery depends on many parameters to ensure stability, prolonged blood circulation, and efficient delivery to the target tissue/organ. Specifically NP surface charge influences the physiochemical stability of NPs and the rate of particle elimination from circulation; previous studies have shown near neutral/slightly anionic NPs have reduced clearance by the reticuloendothelial system^28^,^29^,^30^. Functionalizing the surface of the NP with polymer polyethylene glycol (PEG) is most commonly used to minimize opsonization not only through steric hindrance but also charge shielding^23^,^24^. Since this study focused on evaluating size and time dependent NP accumulation after brain injury, we aimed to minimize the influence of NP parameters outside of size by PEGylating all of our NPs and obtained slightly anionic NPs for efficient systemic delivery.

Passive systemic NP delivery to the injured brain hinges on a damaged BBB and confirmation of a dysfunctional BBB was obtained with the HRP marker (~44 kDa with an estimated diameter of ~3 nm^10^). We observed extravasation of HRP in the primary and the adjacent injury region at 1 h post-injury. However, for all subsequent cohorts, the HRP staining was localized to the primary injury site. A survey of the literature indicates some disagreement in utilizing HRP to classify the underlying mechanisms for BBB breakdown (i.e., injury induced rupture and/or paracellular permeability)^6^,^9^,^31^. As stated previously, the current study focused on evaluating NP accumulation ultimately for acute TBI theranostics. Therefore, it was necessary to correlate NP accumulation with BBB damage through HRP staining. One important observation is that while the incidence of HRP staining reduced over 24 h post injury, significant positive staining was observed out to 24 h after injury indicating the persistence of localized dysfunctional BBB and opportunity for localized NP accumulation. These findings are critical in elucidating the optimal temporal delivery window for NPs (10–1000 nm) as the interest in using NPs for TBI has gained traction recently^21^,^22^. Specifically, two previous studies employed systemic NP delivery after TBI where the NPs ranged from 60 nm to 300 nm in diameter. These studies demonstrated feasibility for NPs to preferentially localize within the injury penumbra when delivered within 4 h post injury^21^,^22^. Yet, little is known about the impact of NP size and injection time to achieve effective delivery after TBI. We directly addressed this critical gap by evaluating a cocktail injection of different sized PEGylated NPs at different time points after injury while maintaining a consistent circulation time (1 h). The peak accumulation for all sized NPs was observed with injections immediately after injury (+1 h circulation time) mirroring the HRP extravasation results. Not surprisingly, we observed prolonged accumulation for two smallest NPs (20 nm and 40 nm) out to 13 h post injury, whereas significant accumulation for the two largest NPs (100 nm and 500 nm) was seen only out to 6 h. Our report is the first to show the evidence of accumulation of up to 500 nm sized PEGylated NPs within the injury penumbra acutely after brain injury. This finding is supported by a NP study on cortical implants in mice where accumulation of up to 500 nm NPs near the implant region was observed at 4 weeks post-implant^32^. Overall, it is evident that our study not only corroborates previous reports, but more importantly expands our current knowledge regarding time and size dependent NP delivery after TBI.

We postulate that TBI pathology directly contributes to NP accumulation within the injury penumbra. TBI, particularly the CCI model, leads to physical rupture of the blood vessels, dysfunction of the BBB and permeable blood vasculature within the injury region^33^,^34^. A similar leaky vasculature phenomenon has been defined as the enhanced permeability and retention (EPR) effect in oncology literature^35^,^36^,^37^,^38^,^39^. Poorly structured and highly permeable vasculature contributes to increased passive accumulation of NPs within solid tumors^36^,^39^,^40^. Thus, the unique pathophysiological nature of the dysfunctional BBB and leaky vasculature after TBI, may lead to localized accumulation of NPs at the injured area due to a similar EPR effect. In the present study we observed localized areas of brain tissue containing multiple sizes of NPs. In contrast, we did not observe significant NP accumulation in uninjured brain tissue as compared to injured tissue, suggesting localized leaky vessels near the injury site. Although the exact mechanism for the NP accumulation at the injury location was not probed, potential mechanisms include accumulation via mechanically-induced ruptured vessels or paracellular diffusion^5^. Future studies are warranted to better understand such mechanisms. Overall, the accumulation of different sized NPs occurred specifically within the primary injury site.

One interesting finding was preferential spatial accumulation within specific cortical regions within the injury penumbra. The CCI injury generated a cortical lesion encompassing the primary motor area (M1), somatosensory area, posterior parietal association area and anteriomedial visual cortex (V1) (from anterior to posterior). Remarkably, at 1 h post-injury significantly higher levels of both HRP and NPs were found within the more anteriorly located primary motor (M1), and somatosensory area compared to posteriorly located parietal association and visual area (V1) of the brain. The specific mechanism that leads to lower accumulation of NPs in the posterior area is not clear. However, we postulate that the heterogeneous nature of the inherent cortical cerebral blood flow and injury-induced alterations in blood flow play key roles. Specifically, regional neural cellular density has been directly correlated with microvessel densities in murine models^41^. Such variations in microvascular density is directly linked to cortical blood flow^42^,^43^. Comparing the cortical regions encompassed in the injury penumbra, we found reports of reduced neural cell density within the parietal cortex^44^. Therefore, the inherently reduced cortical blood flow/microvascular density within the parietal cortex area may largely contribute to a low level of NP accumulation after TBI. Secondarily, TBI promotes localized alterations in the cerebral blood flow, depending on the size and location of contusions and hematomas^45^ leading to abnormal blood supply to injured tissue. The blood supply to the motor, sensory and parietal cortex is supplied by the middle cerebral artery (MCA)^46^. The CCI injury imparted over this region may damage the MCA or its branches resulting in rupture of anteriorly located blood vessels. This type of vascular damage may lead to two phenomena potentially contributing to the regional distribution of NPs, 1. enhanced NP accumulation in anteriorly located blood vessels, and 2. hypoperfusion in downstream posteriorly located regions leading to reduced NP accumulation. Collectively, inherent variations in capillaries combined with injury-induced blood flow alterations may contribute to anteriorly dominate NP accumulation after CCI.

To maximize the NP size spectrum, each animal received an intravenous delivery of a NP cocktail containing four different sized NPs. Our analysis focused on direct comparison within each NP size and did not include cross NP size comparisons. Each NP injection contained an equal mass concentration yet varying number of NPs for each size group, thereby preventing direct comparison across NPs with high fidelity. Each NP group was loaded with a unique fluorophore with discrete fluorescent spectra. Therefore, accumulation of each NP within brain tissue at different time points post-injury was determined through an empirical conversion of total fluorescent intensity specific to each fluorophore to the total number of NPs (Supplemental Fig. 2). Nonetheless, these limitations did not constrain the critical analysis within each NP size group where we revealed never before presented data on the dynamic size NP range delivery after TBI. The results of our study are integral for developing NP-based contrast agents or drug delivery. NPs for brain delivery applications^47^ vary widely in composition ranging from amphiphilic monomers to lipids to more rigid polymer-based^48^,^49^. Smaller NPs (<100 nm) have shown to have slower clearance, higher amount of encapsulated drug accumulation, efficient cellular uptake, and enhanced penetration of poorly permeable tissue, as compared to larger NPs (>200 nm)^40^,^50^,^51^. Previous studies have successfully used NPs (20–60 nm) as indicators of BBB damage in experimental stroke models^52^,^53^ and as theranostic tools for imaging and drug delivery^40^,^51^. Results of our study can potentially be applied to devise multifunctional NPs with therapeutic drugs for brain injury such as superoxide dismutase^54^, erythropoietin^55^, statins^55^ to be loaded into these NPs.

In conclusion, we established that PEGylated polystyrene nanoparticles of different sizes (20 nm, 40 nm, 100 nm and 500 nm) accumulate predominately near the injury region after CCI injury in mice. Furthermore, maximal accumulation for all NP sizes was observed at 1 h post-injury. With a constant circulation time of 1 h across all cohorts, we identified an inverse relationship between the NP size and their accumulation at different time points post injury. The accumulation of NPs was not only influenced by the NP size and time after injury but also varied spatially within the brain tissue cortex. The anterior and middle regions of the injured tissue had maximal accumulation of NPs compared to the posterior region 1 h after brain injury. Detailed studies on biodistribution of each NP and their total accumulation per brain tissue are yet to be addressed. However, our current study provides the groundwork for NP delivery after TBI. Potential application of our study ranges from delivery of targeted contrast agents to therapeutics after TBI. Therefore, better understanding of NP accumulation will facilitate effective utilization of the BBB breakdown for TBI theranostics.

## Materials and Methods

### Materials

Carboxylated polystyrene NPs of different sizes were purchased from Life technologies (Carlsbad, CA, USA). Specifically, 20 nm (F8783), 40 nm (F8793), 100 nm (F8797) and 500 nm (F8813) NPs with dark red (λ_ex_/λ_em_ = 660/680), red (λ_ex_/λ_em_ = 580/605), blue (λ_ex_/λ_em_ = 350/440) and yellow-green (λ_ex_/λ_em_ = 505/515) fluorescence, respectively, were used. Methoxypolyethylene glycol amine 2000 (mPEGamine 2 KDa) (06676), methoxypolyethylene glycol amine 750 (mPEGamine 750 Da) (07966), n-[3-dimethylaminopropyl]-n-ethyl, n-[3-dimethylaminopropyl]-n-ethyl [EDC] (E1769), MES hemisodium buffer (M8902), N-Hydroxysuccinimide (NHS) (56405), and Peroxidase type II from horseradish (P8250-50KU) were purchased from Sigma Aldrich (St. Louis, MO, USA). ImmPACT DAB peroxidase (HRP) substrate (SK-4105) was purchased from Vector laboratories (Burlingame, CA, USA). Slide-A-Lyzer Cassettes (20 K) (66003) were purchased from ThermoFisher scientific (Waltham, MA, USA). Fluorescent mounting media (Vectashield, Vector Labs, Burlingame, Ca, USA)

### Nanoparticle PEG conjugation

Carboxylated NPs were PEGylated using EDC/NHS chemistry. Briefly, mPEGamine 750 Da was mixed with 20 nm NPs (NH_2_:COOH at 2:1 mole excess) whereas mPEGamine 2 kDa was mixed with 40 nm, 100 nm and 500 nm NPs; (NH_2_:COOH at 5:1 mole excess). EDC/NHS (in MES buffer) was added to NP/PEG mixture (8 mM/4 mM for 20 nm and 200 mM/100 mM for other NPs) and HEPES buffer was added to obtain a final pH of 7.8 before incubating for 3 h at room temperature. Glycine (100 mM) was added to quench the reaction. Unbound PEG was removed via dialysis (20 kDa MW). PEGylated NPs were suspended in a 20 mM HEPES (pH 7.4). The concentration of each NP solution was determined with fluorescent standard curves generated from known concentrations of as-received Fluorospheres (FLUOstrar Omega fluorescence plate reader; BMG Labtech, Ortenberg, Germany). Yields of NPs ranged between 40–60%. A concentration of 13.3 mg/ml for each NP was used for all *in vivo* studies.

### Nanoparticle characterization

PEGylated NPs were visualized using transmission electron microscopy (TEM). NPs in water were applied to 300-mesh, carbon coated copper grids for 60 s. After this, excess water was removed by blotting with filter paper before imaging using JEOL 1200EX TEM (Peabody, MA, USA), operated at 80 kV and images were collected with a CCD camera (Scientific Instruments and Accessories; Duluth, GA, USA). The hydrodynamic diameter and zeta potential of NPs in 20 mM HEPES (pH 7.4) were measured pre and post-PEGylation with a dynamic light scattering (DLS) device (Zetasizer Nano Malvern; Malvern, UK). For each NP, three measurements were made and the mean ± standard error of mean (s.e.m.) was reported.

### Controlled cortical impact model

All animal studies were approved by Arizona State University’s Institute of Animal Use and Care Committee (IACUC) and were performed in accordance with the relevant guidelines. Traumatic brain injury (TBI) was modeled using the well-established controlled cortical impact (CCI) injury model^56^. Briefly, adult C57Bl/6 mice (9–10 weeks old) were anesthetized with isoflurane (3% induction, 1.5% maintenance) and placed in stereotaxic frame. The frontoparietal cortex was exposed via 3 mm craniotomy and the impact tip was centered at −1.5 mm bregma and 1.5 mm lateral from midline. The impactor tip diameter was 2 mm, the impact velocity was 6.0 m/s and the depth of cortical deformation was 2 mm and 100 ms impact duration (Impact ONE; Leica Microsystems). The skin was sutured and the animals were placed in a 37 °C incubator until consciousness was regained. The naïve group did not undergo surgery.

### Nanoparticle and horseradish peroxidase (HRP) injection

Retro-orbital injections of the venous sinus in the mouse were performed for intravenous delivery of the particles; this technique is an alternative to tail-vein injection^25^. Animals were anesthetized with isoflurane (3%) and the NP cocktail (75 μl) of different sized NPs (50 mg/kg b.w.) was injected to the right eye, one hour before perfusion and sacrifice. HRP (83 mg/kg b.w.in 25 μl) was injected to the left eye ten mins before perfusion and sacrifice. Depending on the injury group, animals were sacrificed at 1 h, 3 h, 6 h, 13 h, and 24 h post injury. The NP circulation time of 1 h was held constant for each of the cohorts.

### Tissue collection

According to the experimental groups –1 h, 3 h, 6 h, 13 h and 24 h post-injury, animals were deeply anesthetized with lethal dose of sodium pentobarbital solution until a tail pinch produced no reflex movement. Animals were transcardially perfused with cold phosphate-buffered saline (PBS), followed by 4% buffered paraformaldehyde solution. Brain tissue were collected and fixed overnight in 4% buffered paraformaldehyde followed by immersion in 30% sucrose solutions in 1X PBS for cryoprotection for 24 h. Samples were embedded within optimal cutting temperature (OCT) medium and frozen on dry ice. Samples were stored at −80 °C until sectioned coronally at a 25 μm thickness with a cryostat.

### Quantification of nanoparticle accumulation

Slides containing the frozen sections were incubated at room temperature for 20 mins in 1X PBS to rehydrate the tissue and remove OCT compound. Coverslips were mounted on the section after adding one drop of fluorescent mounting media (Vectashield). These sections were imaged using confocal microscopy (Leica TCS SP5 AOBS Spectral Confocal System, 20X magnification). Four region of interest (ROI) of the dimension 775 μm X 775 μm, were selected surrounding the injury penumbra (eight sections per animal, four animals per cohort) and two ROIs at contralateral region (two sections per animal, four animals per cohort). Scanning settings for each NP: 20 nm, 40 nm, 100 nm and 500 nm were λ_ex_/λ_em_ = 633/700–758 nm (800 V gain); λ_ex_/λ_em_ = 561 nm/572–619 nm (645 V gain); λ_ex_/λ_em_ = 405 nm/420–465 nm (585 V gain), and λ_ex_/λ_em_ = 488 nm/507–535 nm (725 V gain), respectively. Configuration settings were maintained constant for all the images collected. For each ROI, Z stacking was performed and total Z width ranged from 20–25 μm with a slice thickness of 1 μm. The Z stacks images were converted to a single image by maximum projection tool using Leica software (LAS AF, Leica microsystems). The sum of four ipsilateral ROI for each section (eight sections per animal, four animals per cohort) were averaged and compared to the sum of the two contralateral ROI (two sections per animal, four animals per cohort). The maximum projected images were thresholded to remove background fluorescence using tissue sections from NP injected naïve brain and total intensity was calculated, using ImageJ software. The fluorescent intensity values were then converted to number of NPs based on an empirical method (See Supplementary Fig. 2).

### Quantification of HRP extravasation

The same tissue section used for NP analysis or their adjacent sections were incubated in PBS buffer for 20 mins. Freshly prepared DAB substrate solution (200 μl) was added and incubated for ten mins at room temperature. Slides were then washed in PBS buffer three times (two mins each) and coverslips were mounted after adding a drop of aqueous mounting media. Sections were imaged using color camera mounted microscope (Leica microscope) at 5X magnification and ROI dimension of 1.50 mm × 2.50 mm were used. ROI were selected surrounding the injury penumbra (eight sections per animal, four animals per cohort) and at contralateral region (two sections per animal, four animals per cohort). The ROI images were then analyzed using ImageJ software (National institute of health, Bethesda, MD, USA) to obtain total number of positive pixels.

### Statistics

Statistical analyses were conducted in GraphPad Prism 5.0 (GraphPad Software, Inc., La Jolla CA). Comparison between zeta potential change of NPs post-PEGylation was done using student’s t-test. Analysis of total positive pixels in ipsilateral and contralateral region of interest for HRP and number of accumulated NP at different time points was conducted using ordinary two-way ANOVA. For HRP and each NP, comparison between ipsilateral ROI and its contralateral ROI was done by student’s t-test. Comparison between ipsilateral ROI for HRP, and individual NPs, over time was conducted using Tukey’s post hoc test. Spatial distribution of HRP extravasation and NP accumulation was done using ordinary two-way ANOVA, followed by Tukey’s post hoc tests.

## Additional Information

**How to cite this article**: Bharadwaj, V. N. *et al*. Temporal assessment of nanoparticle accumulation after experimental brain injury: Effect of particle size. *Sci. Rep.* **6**, 29988; doi: 10.1038/srep29988 (2016).

## Supplementary Material

Supplementary Information

## Figures and Tables

**Table 1:** Nanoparticle characterization: Hydrodynamic diameter and zeta potential of non-PEGylated NP and PEGylated NP, mean ± standard error of mean (n = 3).

**Nominal NP size (nm)**	**Hydrodynamic Diameter (nm) of NP**		**Zeta Potential (mV) of NP**	
	**non-PEGylated**	**PEGylated**	**non-PEGylated**	**PEGylated**
20	19.6 ± 2.0	24.7 ± 2.0	−44.1 ± 3.3	−28.4 ± 3.2*
40	50.1 ± 3.5	58.4 ± 4.0	−46.0 ± 3.5	−9.2 ± 4.0*
100	91.5 ± 5.4	101.9 ± 6.0	−55.3 ± 3.1	−22.4 ± 3.5*
500	507.0 ± 27.5	517.6 ± 34.8	−57.9 ± 1.5	−26.5 ± 2.0*

*p < 0.05, t-test. Measurements in 20 mM HEPES (pH 7.4).
