# Supplementary material for: Temporal assessment of nanoparticle accumulation after experimental brain injury: Effect of particle size
Source: Sci Rep. 2016 Jul 22;6:29988. doi: 10.1038/srep29988 (PMC4957235; doi:10.1038/srep29988)
Supplement: Supplementary Information [file srep29988-s1.pdf]

Supplementary

Title: Temporal assessment of nanoparticle accumulation after brain injury: Effect of particle size

Authors: Vimala N. Bharadwaj, Jonathan Lifshitz, P. David Adelson, Vikram D. Kodibagkar, Sarah E. Stabenfeldt

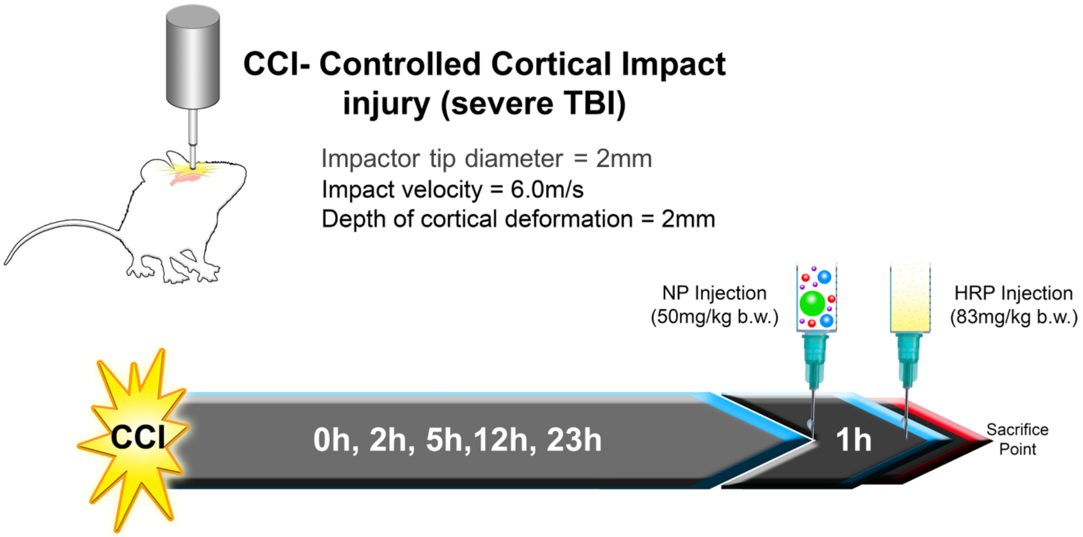

Figure S1. ***In vivo*** **experimental study design:** Cocktail of different size nanoparticles (NP: 20 nm, 40 nm, 100 nm and 500 nm) was injected intravenously at various time points post CCI and animals were sacrificed one hour post injection. HRP was injected intravenously, 10 min before sacrifice.

Calibration curve for fluorescent intensity vs number of NPs

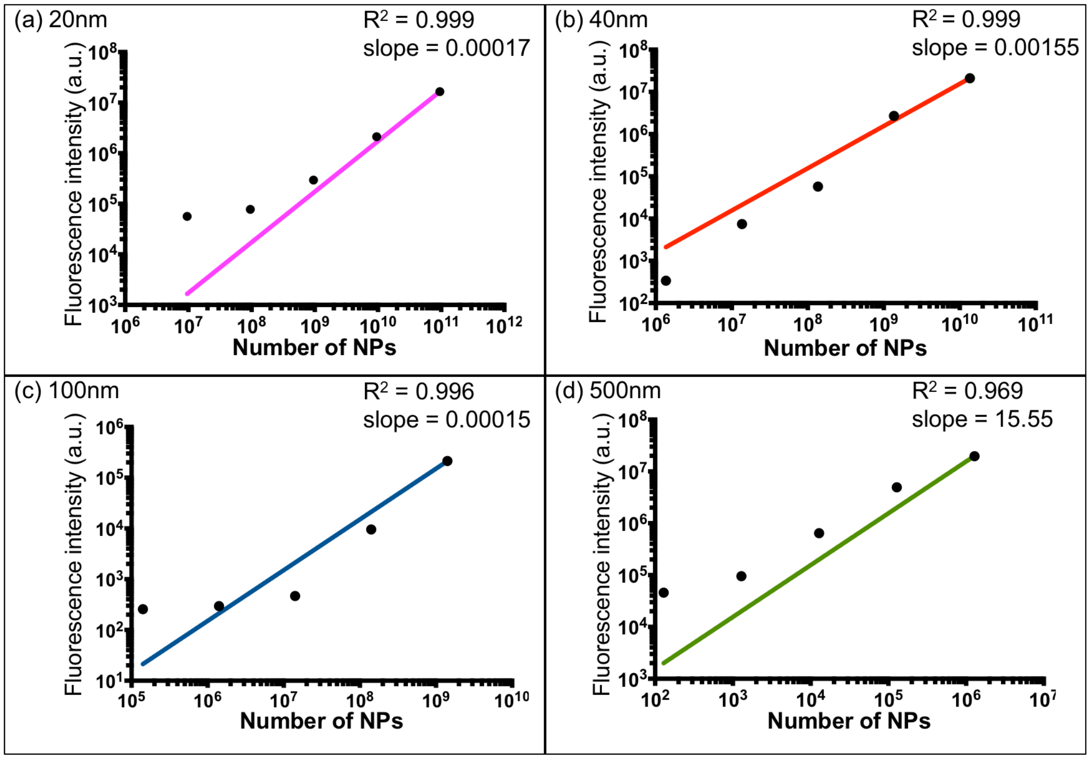

Figure S2. Calibration curves for each NPs. (a) – (d) Linear fit calibration curves for 20 nm, 40 nm, 100 nm and 500 nm NP (a.u. arbitrary units). Data (n =5) and fits presented on a log-log scale for clarity, with R^2 ranging from 0.969 – 0.999.

Known concentrations of NPs with five dilutions: 1333 µg/ml, 133.3 µg/ml, 13.33 µg/ml, 1.333 µg/ml, and 0.1333 µg/ml were used, in triplicates. 1ul of solution was carefully pipetted into hemocytometer and measured using 20X magnification of confocal microscope. The confocal microscopy parameters were kept consistent with that used for the brain tissue sections. Two regions for each four of the channels were measured in three different samples. The amount of solution in these regions was determined by dividing the total volume of solution added and total number of squares on hemocytometer. The amount of solution (µg) was then converted to the number of NPs using the information provided by

the manufactures for each NP size. A calibration curve with the intensity on y-axis and number of NP on x-axis was plotted (Figure S2). Each NP showed linear trend, with R^2 ranging between 0.96-0.99. The reciprocal of slopes obtained from each curve was multiplied with the intensity obtained for the brain tissue measurement, to calculate the number of each NP.
